# Clinical characteristics of external bacterial ocular and periocular infections and their antimicrobial treatment patterns among a Ghanaian ophthalmic population

**DOI:** 10.1038/s41598-022-14461-x

**Published:** 2022-06-17

**Authors:** Isaiah Osei Duah Junior, Michel Pascal Tchiakpe, Lawrence Sheringham Borquaye, Kwadwo Amoah, Francis Kwaku Dzideh Amankwah, David Ben Kumah, Linda Aurelia Ofori, Anthony Danso-Appiah, Bright Owusu Prempeh, Stephen Yao Gbedema, Justin Munyaneza, Cynthia Amaning Danquah, Kwadwo Owusu Akuffo

**Affiliations:** 1 Department of Optometry and Visual Science, College of Science, Kwame Nkrumah University of Science and Technology, Kumasi, Ghana; 2 Department of Chemistry, College of Science, Kwame Nkrumah University of Science and Technology, Kumasi, Ghana; 3 Central Laboratory, Kwame Nkrumah University of Science and Technology, Kumasi, Ghana; 4 The Eye Clinic, Kumasi South Hospital, Atonsu-Agogo, Kumasi, Ghana; 5 Department of Pharmaceutics, Faculty of Pharmacy and Pharmaceutical Sciences, College of Health Sciences, Kwame Nkrumah University of Science and Technology, Kumasi, Ghana; 6 Department of Theoretical and Applied Biology, College of Science, Kwame Nkrumah University of Science and Technology, Kumasi, Ghana; 7 Department of Epidemiology and Disease Control, School of Public Health, University of Ghana, Legon, Ghana; 8 University of Ghana Centre for Evidence Synthesis and Policy, School of Public Health, University of Ghana, Legon, Ghana; 9 The Anglican Eye Hospital, Jachie, Ghana; 10 Department of Pharmacology, Faculty of Pharmacy and Pharmaceutical Sciences, College of Health Sciences, Kwame Nkrumah University of Science and Technology, Kumasi, Ghana

**Keywords:** Microbiology, Diseases

## Abstract

Empirical antimicrobial therapy is linked to a surge in antimicrobial resistant infections. However, an insight on the bacteria etiology of ocular infections is essential in the appropriation of choice of antimicrobial among clinicians, yet there remains a dearth of data from Ghana. We investigated the bacteria etiology of external ocular and periocular infections and antimicrobial treatment patterns among a Ghanaian ophthalmic population. A multicenter study design with purposive sampling approach was employed. Patients demographics and clinical data were collated using a pretested structure questionnaire. Cornea specimens and conjunctival swabs were obtained for bacterial isolation following standard protocols. About 95% (98/103) of ocular samples were positive for bacteria culture. The proportion of Gram-negative bacteria was 58.2%, and the predominant bacteria species were *Pseudomonas aeruginosa* 38.8% and *Staphylococcus aureus* 27.6%. Conjunctivitis 40.0% and keratitis 75.0% were mostly caused by *Pseudomonas aeruginosa*. The routinely administered antimicrobial therapy were polymyxin B 41.2%, neomycin 35.1% and ciprofloxacin 31.6%. Participants demographic and clinical characteristics were unrelated with positive bacteria culture (p > 0.05). Our results showed a markedly high burden of ocular bacterial infections and variations in etiology. Bacterial infection-control and antimicrobial agent management programs should be urgently institutionalized to prevent the emergence of resistant infections.

## Introduction

Globally, eye infections of bacterial origin remain a significant contributor to ocular morbidity and blindness, and the burden is increasing^1,2^. Further, results from ocular microbial studies across different populations show no obvious pattern in prevalence estimates (ranging from 21.8 to 82.5%) across Africa^2–12^, Asia^13–20^, Australia^21,22^, Europe^23–25^ and North America^26^. Similarly, the bacterial etiology, thus the nature of Gram bacteria and the species of bacteria commonly implicated in external ocular (surface of the eyeball) and periocular (surrounding of the eyeball) infections vary across geographical regions and settings^2–13,15–20,22,24–26^.

Anatomically, the eye is divided into three tunics: the outer (conjunctiva, sclera, cornea), middle (lens, ciliary body, iris), and inner (retina) coats. The tear film contains innate defenses such as bacteriocin, beta-lysin, lipocalin, lysozymes, immunoglobulins (Ig A, Ig G, Ig M), lactoferrins, and with antimicrobial effect against pathogenic strain of microorganisms. The external milieu of the eyes serves as a biome for pathogenic and non-pathogenic organisms. Naturally, antimicrobial constituents within the tear film prevent opportunistic microbes from causing any infections to the eyes. Dysregulation of the homeostatic balance as a result of trauma, contact lens wear, surgery, use of topical antibiotics, and reduced systemic immunity predisposes the eyes to opportunistic pathogens such as bacteria, fungi, virus, and protozoa. However, among these microbes, bacteria are commonly implicated in external ocular and periocular infections^27^. Based on the exterior region affected; external ocular infections can be classified as blepharitis, conjunctivitis, keratitis, dacryocystitis, preseptal and orbital cellulitis^28–30^, thus eyelids, conjunctiva, cornea, lacrimal sac, pre and post-septal areas are respectively involved.

These eye infections are usually treated with broad-spectrum antimicrobial agents with varying modes of action without any proper follow up for culture and sensitivity testing to identify the implicated pathogen^31^. In resource limited-environments such as Ghana, due to the unavailability of rapid diagnostic testing facilities, especially for identifying fastidious organisms, most clinicians: including eye care professionals engage in this prevailing empirical broad-spectrum antimicrobial treatment therapy^32^. Consequently, the irrational and prolonged use of broad-spectrum antibiotics in treating eye infections could alter the genetic makeup of ocular bacteria and consequently lead to antimicrobial resistance^33^.

Ocular antimicrobial resistance is a growing public health threat in both advanced and developing countries. In the developed world nationwide surveillance programs have been institutionalized to monitor ascendancies in the antimicrobial resistance curve and subsequently tackle it. Among these nationwide surveillance programs include Ocular Tracking Resistance in the U.S. Today (Ocular TRUST)^34^, Antibiotic Resistance Monitoring in Ocular Microorganisms (ARMOR)^35^, European Antimicrobial Resistance Surveillance System^36^, and Swedish Strategic Programme for the Rational Use of Antimicrobial Agents and Surveillance of Resistance^37^. However, in developing countries such as Ghana, aside from the limited accessibility to a national antimicrobial policy, there are compromise regulatory measures and poor adherence to the use of antibiotics^38^.

Multidrug resistance (MDR) is gradually gaining attention in mainstream medicine and public healthcare generally, as it renders antimicrobial agents inefficacious against pathogenic strains of bacteria. MDR causes delays in treatment and recoveries, rise in cost of therapy as well as increase in hospitalization time^39–41^. In a nationwide laboratory based surveillance studies in Ghana, Opintan et al. reported over 70% prevalence of MDR among antibiotics such chloramphenicol, gentamycin, tetracycline, and quinolones against isolated bacterial strains from various infections of the urine, blood, sputum, ears, and eyes^42^. This finding was consistent with studies conducted in the People’s Republic of China^43,44^, Italy^45^, and Ethiopia^12^ which showed similar increasing trends. Consequentially, without drastic measures, it is estimated that the world will experience over 10 million annual AMR-related deaths hence it has become imperative to devise ammunitions to curb the situation^46^. Importantly, a decline in ocular MDR will result in increased life expectancy as ocular resistance infections and associated blindness induce mortality.

There is a paucity of data on the prevalence and bacteria etiology of ocular infections in Ghana^9,27^. Furthermore, earlier microbiological investigations in Ghana did not exquisitely focus on ocular infections and associated microbes^42,47–49^. The absence of country-specific contemporary estimates limit the modeling of future scenarios, and assumptions with unreliable data and/or making decisions with evidence from other countries is of questionable utility given the geographic differences. An insight on bacteria etiology of ocular infections presented by Ghanaian patients is critical for desirable choice of antibiotic therapy by clinicians. Therefore, the study aims to investigate the bacteria etiology of external ocular and periocular infections, and antimicrobial treatment patterns among a Ghanaian ophthalmic population. The isolates recovered from ocular specimen will aid future antibiotic sensitivity studies and also serve as a gateway for exploration of local medicinal plants as alternative therapeutic agents.

## Results

### Description of the sample

Table 1 presents the sociodemographic, socioeconomic and healthcare status characteristics of the study participants. Out of the 114 patients presenting with external ocular and periocular infections, majority were females (56.1%), of median age of 17.0 (Interquartile range; 29.75) years (Table 1). The majority of the participants were aged 3–17 years (29.8%), of Akan ethnicity (93.0%), and with a protestant religion (80.7%). Most of them lived in a rural community (59.6%), with their highest education level being primary (33.6%) and major occupation as students (43.9%). An equal proportion were single (21.2%) and married (21.2%) and the remaining either cohabiting (3.5%), divorced (2.7%), widow (1.8%) or separated (0.9%). A preponderance of participants never smoked (52.6%), and with a significantly higher average alcohol intake in males compared to females (p = 0.027). Approximately 6% had hypertension and an equal proportion had diabetes (4.4%) and peptic ulcer (4.4%). The number of patients on antihypertensive, antidiabetic and antibiotic medications were 5.3%, 3.5% and 3.5%, respectively.

**Table 1:** Description of the sample.

Variables	Total (N = 114)	Males (N = 50, 43.9%)	Females (N = 64, 56.1%)	*p*-value for linearity
	% (frequency)	% (frequency)	% (frequency)	
***Demographic characteristics***				
**Age group (years)**				
0–2	21.1 (24)	20.0 (10)	21.9 (14)	0.948
3–7	29.8 (34)	32.0 (16)	28.1 (18)	
18–39	27.2 (31)	28.0 (14)	26.6 (17)	
≥ 40	21.9 (25)	20.0 (10)	23.4 (15)	
**Ethnicity**^**‡**^				
Akan	93.0 (106)	90.0 (45)	95.3 (61)	0.271
Northerner	7.0 (8)	10.0 (5)	4.7 (3)	
**Religion**				
Catholic	13.2 (15)	22.0 (11)	6.3 (4)	0.041
Protestant	80.7 (92)	76..0 (38)	84.4 (54)	
Muslim	4.4 (5)	2.0 (1)	6.3 (4)	
Atheist	1.8 (2)	0.0 (0)	3.1 (2)	
***Socioeconomic characteristics***				
**Residence**				
Rural	59.6 (68)	60.0 (30)	59.4 (38)	0.946
Urban	40.4 (46)	40.0 (20)	40.6 (26)	
**Highest level of education**				
None	4.4 (5)	4.0 (2)	4.8 (3)	0.943
Preschool	13.3 (15)	12.0 (6)	14.3 (9)	
Primary	33.6 (38)	30.0 (15)	36.5 (23)	
Secondary	29.2 (33)	32.0 (16)	27.0 (17)	
Tertiary	8.0 (9)	10.0 (5)	6.3 (4)	
Not applicable*	11.5 (13)	12.0 (6)	11.1 (7)	
**Occupation**				
Farming	4.4 (5)	6.0 (3)	3.1 (2)	0.092
Wage/Salary worker	7.0 (8)	6.0 (3)	7.8 (5)	
Construction worker	2.6 (3)	6.0 (3)	0.0 (0)	
Dressmaking	1.8 (2)	0.0 (0)	3.1 (2)	
Driver/Transport business	2.6 (3)	6.0 (3)	0.0 (0)	
Businessman/woman	5.3 (6)	4.0 (2)	6.3 (4)	
Welding	1.8 (2)	4.0 (2)	0.0 (0)	
Trading	8.8 (10)	4.0 (2)	12.5 (8)	
Beautician	1.8 (2)	0.0 (0)	3.1 (2)	
Hairdressing	2.6 (3)	2.0 (1)	3.1 (2)	
Student	43.9 (50)	46.0 (23)	42.2 (27)	
Unemployed	5.3 (6)	2.0 (1)	7.8 (5)	
Not applicable*	12.3 (14)	14.0 (7)	10.9 (7)	
**Marital status**				
Not applicable*	48.7 (55)	50.0 (25)	47.6 (30)	0.936
Single	21.2 (24)	24.0 (12)	19.0 (12)	
Married	21.2 (24)	18.0 (9)	23.8 (15)	
Cohabiting	3.5 (4)	4.0 (2)	3.2 (2)	
Divorced	2.7 (3)	2.0 (1)	3.2 (2)	
Separated	0.9 (1)	0.0 (0)	1.6 (1)	
Widow	1.8 (2)	2.0 (1)	1.6 (1)	
***Health status variables***				
**Smoking habits**				
Not applicable*	44.7 (51)	46.0 (23)	43.8 (28)	0.237
Never smoked	52.6 (60)	48.0 (24)	56.3 (36)	
Past smoker	1.8 (2)	4.0 (2)	0.0 (0)	
Current smoker	0.9 (1)	2.0 (1)	0.0 (0)	
**Alcohol intake habits**				
Not applicable*	43.9 (50)	42.0 (21)	45.3 (29)	0.054
I never drink	46.5 (53)	40.0 (20)	51.6 (33)	
I drink only on special occasions	7.9 (9)	14.0 (7)	3.1 (2)	
I drink once or twice a week	1.8 (2)	4.0 (2)	0.0 (0)	
**Average alcohol consumption per week**				
None	90.4 (103)	82.0 (41)	96.9 (62)	0.027
1 unit	6.1 (7)	12.0 (6)	1.6 (1)	
2–5 unit	3.5 (4)	6.0 (3)	1.6 (1)	
**Medical history**				
Diabetes	4.4 (5)	2.0 (1)	6.3 (4)	0.272
Hypertension	6.1 (7)	4.0 (2)	7.8 (5)	0.4
Tuberculosis	0.9 (1)	0.0 (0)	1.6 (1)	0.375
Sexually transmitted diseases	0.9 (1)	2.0 (1)	0.0 (0)	0.256
Peptic ulcer	4.4 (5)	2.0 (1)	6.3 (4)	0.272
Others	2.6 (3)	6.0 (3)	0.0 (0)	0.047
**Is condition ongoing**				
Not applicable*	80.7 (92)	82.0 (41)	79.7 (51)	0.915
No	2.6 (3)	2.0 (1)	3.1 (20	
Yes	16.7 (19)	16.0 (8)	17.2 (11)	
**Are you currently taking any medication**				
No	82.5 (94)	82.0 (41)	82.8 (53)	0.91
Yes	17.5 (20)	18.0 (9)	17.2 (11)	
**Class of medications**				
Antibiotic	3.5 (4)	6.0 (3)	1.6 (1)	0.201
Antidiabetic	3.5 (4)	2.0 (1)	4.7 (3)	0.439
Antihypertensive	5.3 (6)	4.0 (2)	6.3 (4)	0.593
Antimalarial	0.9 (1)	0.0 (0)	1.6 (1)	0.375
Anticholesterol	0.9 (1)	2.0 (1)	0.0 (0)	0.256
Others	7.9 (9)	8.0 (4)	7.8 (5)	0.971

*Under age; %, percentage frequency.

^‡^Northerner is a collective name for all ethnic group in the northern region of Ghana.

### Clinical characteristics of external ocular and periocular infections among study participants

Table 2 shows the clinical characteristics of external ocular and periocular infections among study participants. Most presented with both eyes infected (66.7%) with the conjunctiva (94.7%) as the commonest affected site. The majority of the participants had previously used antimicrobials (55.8%) and have had previous eye infections (45.1%), and with duration of infection less than one-week (63.7%). There was a significant variation between males and females in terms of previous ocular trauma (p = 0.023) and previous use of mascara (p = 0.015). With respect to monocular visual acuity, majority had right eye visual acuity better than 6/18 (63.8%) and with a fewer having visual acuity worse than 6/60 (2.6%). Most had a left visual acuity within the ranges of 6/5–6/6 (67.5%) and fewer proportion (1.8%) having visual acuity better than 6/60 but worse than 6/24. The commonest presenting symptom was hyperemia/redness (69.3%), followed by discharge, (62.3%) itching (60.5%), eye pain (50.9%) and a smaller fraction having falling lashes (0.9%). Majority received a minimum of two antibiotics for treatment of infections (40.4%) and with a fewer portion (7.9%) having treatment other than antibiotics. The proportion of clinical presentations were conjunctivitis (60.5%), keratoconjunctivitis (11.4%), blepharoconjunctivis (9.6%), keratitis (7.9%), ocular trauma (6.1%), hordeolum (2.6%), preseptal cellulitis (0.9%) and ophthalmia neonatorum (0.9%). Other associated conditions were dry eyes (10.5%), headaches (5.3%), pterygium (4.4%) and pinguecula (0.9%). The commonly used antimicrobial therapeutics were polymyxin B (41.2%), neomycin (35.1%) and ciprofloxacin (31.6%) and fewer instances gentamycin (2.6%) and ofloxacin (1.6%) as shown in Table 3.

**Table 2:** Clinical characteristics of external ocular and periocular infections among a Ghanaian ophthalmic population.

	Total (N = 114)	Males (N = 50, 43.9%)	Females (N = 64, 56.1%)	*p*-value for linearity
	% (frequency)	% (frequency)	% (frequency)	
**Eyes affected**				
Right eye; oculus dexter	16.7 (19)	22.0 (11)	12.5 (8)	0.369
Left eye; oculus sinister	16.7 (19)	14.0 (7)	18.8 (12)	
Both eyes; oculus uterque	66.7 (76)	64.0 (32)	68.8 (44)	
**Site of eye affected**				
Eyelid/eye lashes	20.2 (23)	22.0 (11)	18.8 (12)	0.668
Conjunctiva	94.7 (108)	92.0 (46)	96.9 (62)	0.247
Cornea	18.4 (21)	26.0 (13)	12.5 (8)	0.065
**Risk factors**				
Previous eye infections	45.1 (51)	44.0 (22)	46.0 (29)	0.829
Previous use of antimicrobials	55.8 (63)	52.0 (26)	58.7 (37)	0.474
Previous usage of contact lenses	0.9 (1)	0.0 (0)	1.6 (1)	0.371
Previous use of spectacles	9.7 (11)	10.0 (5)	9.5 (6)	0.932
Previous ocular trauma	20.4 (23)	30.0 (15)	12.7 (8)	0.023
Previous ocular surgery	2.7 (3)	2.0 (1)	3.2 (2)	0.700
**Duration of illness**				
< 1 week	63.7 (72)	66.0 (33)	61.9 (39)	0.649
2–4 weeks	18.6 (21)	20.0 (10)	17.5 (11)	
> 4 weeks	17.7 (20)	14.0 (7)	20.6 (13)	
Previous application of mascara	6.2 (7)	0.0 (0)	11.1 (7)	0.015
Previous application of breastmilk	2.7 (3)	2.0 (1)	3.2 (2)	0.700
***Presenting visual acuity***				
**Right eye; oculus dexter**				
Unavailable	2.6 (3)	2.0 (1)	3.1 (2)	0.939
FFL	23.7 (27)	24.0 (12)	23.4 (15)	
6/5–6/18	63.2 (72)	62.0 (31)	64.1 (41)	
6/24–6/60	7.9 (9)	8.0 (4)	7.8 (5)	
3/60–1/60	2.6 (3)	4.0 (2)	1.6 (1)	
**Left eye; oculus dexter**				
Unavailable	2.6 (3)	2.0 (1)	3.1 (2)	0.356
FFL	23.7 (27)	24.0 (12)	23.4 (15)	
6/5–6/18	67.5 (77)	66.0 (33)	68.8 (44)	
6/24–6/60	1.8 (2)	0.0 (0)	3.1 (2)	
3/60–1/60	4.4 (5)	8.0 (4)	1.6 (1)	
**Both eyes; oculus uterque**				
Unavailable	91.2 (104)	86.0 (43)	95.3 (61)	0.266
FFL	0.9 (1)	2.0 (1)	0.0 (0)	
6/5–6/18	7.0 (8)	10.0 (5)	4.7 (3)	
3/60–1/60	0.9 (1)	2.0 (1)	0.0 (0)	
**Presenting patient symptoms**				
Eye pain	50.9 (58)	42.0 (21)	57.8 (37)	0.094
Itching	60.5 (69)	54.0(27)	65.6 (42)	0.208
Falling lashes	0.9 (1)	0.0 (0)	1.6 (1)	0.375
Lacrimation/watering	61.4 (70)	64.0 (32)	59.4 (38)	0.615
Hyperemia/redness	69.3 (79)	72.0 (36)	67.2 (43)	0.580
Swelling	21.9 (25)	22.0 (11)	21.9 (14)	0.987
Discharge	62.3 (71)	70.0 (35)	56.3 (33)	0.133
Burning sensation	7.9 (9)	6.0 (3)	9.4 (6)	0.507
Foreign body sensation	10.5 (12)	14.0 (7)	7.8 (5)	0.285
Others	11.4 (13)	4.0 (2)	17.2 (11)	0.028
**Test/investigations**				
Visual acuity	99.1 (113)	98.0 (49)	100 (64)	0.256
Slit lamp Biomicroscopy	99.1 (113)	98.0 (49)	100 (64)	0.256
Ophthalmoscopy	98.2 (112)	98.0 (49)	98.4 (63)	0.860
Microbial analysis	90.4 (103)	88.0 (44)	92.2 (59)	0.452
***Clinical signs***				
**Eyelashes**				
Healthy	98.2 (112)	100.0 (50)	96.9 (62)	0.451
Misdirected	0.9 (1)	0.0 (0)	1.6 (1)	
Crust formation	0.9 (1)	0.0 (0)	1.6 (1)	
**Eyelids**				
Healthy	66.7 (76)	66.0 (33)	67.2 (43)	0.819
Papillae	17.5 (20)	18.0 (9)	17.2 (11)	
Cobblestones	1.8 (2)	2.0 (1)	1.6 (1)	
Swelling	11.4 (13)	12.0 (6)	10.9 (7)	
Crust	0.9 (1)	2.0 (1)	0.0 (0)	
Drooping	0.9 (1)	0.0 (0)	1.6 (1)	
Rashes	0.9 (1)	0.0 (0)	1.6 (1)	
**Conjunctiva**				
Healthy	26.3 (30)	28.0 (14)	25.0 (16)	0.646
Injection	71.9 (82)	72.0 (36)	71.9 (46)	
Limbal papillae	0.9 (1)	0.0 (0)	1.6 (1)	
Limbal pigmentation	0.9 (1)	0.0 (0)	1.6 (1)	
**Cornea**				
Transparent	87.7 (100)	78.0 (39)	95.3 (61)	0.045
Opacities	3.5 (4)	4.0 (2)	3.1 (2)	
Laceration	0.9 (1)	2.0 (1)	0.0 (0)	
Abrasions	7.0 (8)	14.0 (7)	1.6 (1)	
Ulcer	0.9 (1)	2.0 (1)	0.0 (0)	
**Sclera**				
Healthy	83.3 (95)	78.0 (39)	87.5 (56)	0.177
Pigmented	16.7 (19)	22.0 (11)	12.5 (8)	
**Anterior chamber**				
Deep	98.2 (112)	96.0 (48)	100 (64)	0.106
Shallow	1.8 (2)	4.0 (2)	0.0 (0)	
**Pupils**				
Healthy (PERLLA)	99.1 (113)	98.0 (49)	100.0 (64)	0.256
Abnormal (RAPD)	0.9 (1)	2.0 (1)	0.0 (0)	
**Iris**				
Health (Dark and flat)	99.1 (113)	98.0 (49)	100.0 (64)	0.256
Prolapse	0.9 (1)	2.0 (1)	0.0 (0)	
**Lens**				
Transparent	94.7 (108)	92.0 (46)	96.9 (62)	0.247
Opacities	5.3 (6)	8.0 (4)	3.1 (2)	
**Number of antibiotics administered**				
None	7.9 (9)	6.0 (3)	9.4 (6)	0.351
1	33.3 (38)	38.0 (19)	29.7 (19)	
2	40.4 (44)	44.0 (22)	37.5 (24)	
≥ 3	18.4 (21)	12.0 (6)	23.4 (15)	
**Clinical presentation**				
Conjunctivitis	60.5 (69)	52.0 (26)	67.2 (43)	0.216
Blepharoconjuntivitis	9.6 (11)	10.0 (5)	9.4 (6)	
Keratoconjunctivitis	11.4 (13)	16.0 (8)	7.8 (5)	
Ophthalmia neonatorum	0.9 (1)	2.0 (1)	0.0 (0)	
Ocular trauma	6.1 (7)	8.0 (4)	4.7 (3)	
Preseptal cellulitis	0.9 (1)	0.0 (0)	1.6 (1)	
Hordeolum	2.6 (3)	0 (0.0)	3 (4.7)	
Keratitis	7.9 (9)	6 (12.0)	3 (4.7)	
**Associated conditions**				
Dry eye syndrome	10.5 (12)	6.0 (3)	14.1 (9)	0.164
Pterygium	4.4 (5)	4.0 (2)	4.7 (3)	0.859
Pingueculum	0.9 (1)	0.0 (0)	1.6 (1)	0.375
Headaches	5.3 (6)	8.0 (4)	3.1 (2)	0.247

*FFL* fixate-and-follow light, *PERLLA* pupils are equal, round, and reactive to light and accommodation, *RAPD* relative afferent pupillary defect.

**Table 3:** Antimicrobial treatment of external ocular and periocular infections in a Ghanaian ophthalmic population.

Types of clinical presentation	Bacteria culture results n (%)		Antimicrobial therapy employed in treating external ocular and periocular infections n (%)									
	Positive	Negative	RFR	CIP	GTM	PoB	NM	TBM	TX	OTX	OFC	FX
Conjunctivitis	55 (94.8)	3 (5.2)	0 (0.0)	22 (31.9)	2 (2.9)	29 (42.0)	22 (31.9)	13 (18.8)	16 (23.2)	12 (2.6)	0 (0.0)	0 (0.0)
Blepharoconjuntivitis	11 (100.0)	0 (0.0)	0 (0.0)	2 (18.2)	0 (0.0)	8 (72.7)	5 (45.5)	1 (9.1)	2 (18.2)	3 (27.3)	0 (0.0)	0 (0.0)
Keratoconjunctivitis	12 (92.3)	1 (7.7)	0 (0.0)	2 (15.4)	0 (0.0)	5 (38.5)	9 (69.2)	0 (0.0)	4 (30.8)	0 (0.0)	0 (0.0)	0 (0.0)
Ophthalmia neonatorum	1 (100.0)	0 (0.0)	0 (0.0)	0 (0.0)	0 (0.0)	1 (100.0)	0 (0.0)	1 (100.0)	0 (0.0)	1 (100.0)	0 (0.0)	0 (0.0)
Ocular trauma	7 (100.0)	0 (0.0)	1 (14.3)	3 (42.9)	0 (0.0)	1 (14.3)	1 (14.3)	0 (0.0)	2 (28.6)	0 (0.0)	0 (0.0)	1 (14.3)
Preseptal cellulitis	1 (100.0)	0 (0.0)	0 (0.0)	0 (0.0)	0 (0.0)	0 (0.0)	1 (100.0)	0 (0.0)	1 (100.0)	0 (0.0)	0 (0.0)	1 (100.0)
Hordeolum	3 (100.0)	0 (0.0)	0 (0.0)	1 (33.3)	0 (0.0)	1 (33.3)	1 (33.3)	0 (0.0)	0 (0.0)	0 (0.0)	0 (0.0)	3 (100.0)
Keratitis	8 (88.9)	1 (11.1)	0 (0.0)	6 (66.7)	1 (11.1)	2 (22.2)	1 (11.1)	1 (11.1)	3 (33.3)	2 (22.2)	1 (11.1)	0 (0.0)
Total	98 (95.1)	5 (4.4)	1 (0.9)	36 (31.6)	3 (2.6)	47 (41.2)	40 (35.1)	16 (14.0)	28 (24.6)	18 (15.8)	1 (0.9)	5 (4.4)

*n* frequency, % percentage frequency, *RFR* referral, *CIP* ciprofloxacin, *GTM* gentamycin, *PoB* polymyxin B, *NM* neomycin, *TBM* tobramycin, *TX* tetracycline, *OXT* oxytetracycline, *OFC* ofloxacin, *FX* flucoxacillin .

### Bacteria etiology of external ocular and periocular infections among study participants

One hundred and three (103/114) ocular specimens were enrolled for bacterial isolation owing to the inability to obtain swabs from uncooperative minor subjects. Ninety-eight (95.1%) of the samples were culture positive, and no mixed cultured was identified (Please see Table 3). The proportion of Gram-negative bacteria was 58.2% with *Pseudomonas aeruginosa* (38.8%) and *Pseudomonas putida* (11.2%) being the predominant species. Conversely, the prevalence of Gram-positive bacteria was 41.8%, with a preponderance of bacteria pathogens being *Staphylococcus aureus* (27.6%) and *Coagulase negative staphylococci, CONS* (13.3%). The commonest strains of bacteria pathogens isolated from conjunctivitis were *Pseudomonas aeruginosa* (40.0%)*, Staphylococcus aureus* (21.8%) and *Pseudomonas putida* (16.4%). Similarly, *Pseudomonas aeruginosa* (41.7%) and *Staphylococcus aureus* (33.3%) were frequently identified in cases of keratoconjunctivitis. *Staphylococcus aureus* (100.0%) was the sole organism implicated in cases of preseptal cellulitis, whereas most cases of keratitis was caused by *Pseudomonas aeruginosa* (75.0%) as shown in Table 4.

**Table 4:** Distribution of bacteria isolates across different clinical presentations of external ocular and periocular infections in a Ghanaian ophthalmic population.

Bacteria isolates	Types of clinical presentation								Total isolates n (%)
	Conjunctivitis n (%)	Blepharoconjuntivitis n (%)	Keratoconjunctivitis n (%)	Ophthalmia neonatorum n (%)	Ocular trauma n (%)	Preseptal cellulitis n (%)	Hordeolum n (%)	Keratitis n (%)	
**Gram positive**									
*S. aureus*	12 (21.8)	8 (72.7)	4 (33.3)	0 (0.0)	1 (14.3)	1 (100.0)	0 (0.0)	1 (12.5)	27 (27.6)
*CONS*	8 (14.5)	2 (18.2)	1 (8.3)	1 (100.0)	1 (14.3)	0 (0.0)	0 (0.0)	0 (0.0)	13 (13.3)
*Streptococcus spp*	1 (1.8)	0 (0.0)	0 (0.0)	0 (0.0)	0 (0.0)	0 (0.0)	0 (0.0)	0 (0.0)	1 (1.0)
**Gram negative**									
*Citrobacter spp*	1 (1.8)	0 (0.0)	0 (0.0)	0 (0.0)	1 (14.3)	0 (0.0)	0 (0.0)	0 (0.0)	2 (2.0)
*Serratia spp*	0 (0.0)	0 (0.0)	0 (0.0)	0 (0.0)	0 (0.0)	0 (0.0)	1 (33.3)	0 (0.0)	1 (1.0)
*Salmonella spp*	1 (1.8)	0 (0.0)	1 (8.3)	0 (0.0)	1 (14.3)	0 (0.0)	0 (0.0)	0 (0.0)	3 (3.1)
*E. coli*	1 (1.8)	0 (0.0)	0 (0.0)	0 (0.0)	0 (0.0)	0 (0.0)	0 (0.0)	0 (0.0)	1 (1.0)
*Klebsiella spp.*	0 (0.0)	0 (0.0)	0 (0.0)	0 (0.0)	1 (14.3)	0 (0.0)	0 (0.0)	0 (0.0)	1 (1.0)
*P. aeruginosa*	22 (40.0)	1 (9.1)	5 (41.7)	0 (0.0)	2 (28.6)	0 (0.0)	2 (66.7)	6 (75.0)	38 (38.8)
*P. putida*	9 (16.4)	0 (0.0)	1 (8.3)	0 (0.0)	0 (0.0)	0 (0.0)	0 (0.0)	1 (12.5)	11(11.2)
Total	55 (56.1)	11 (11.2)	12 (12.2)	1 (1.0)	7 (7.1)	1 (1.0)	3 (3.1)	8 (8.2)	98 (100.0)

*n* frequency, % percentage frequency, *CONS* coagulase-negative staphylococcal species.

### Factors associated with external ocular and periocular infections

Table 5 shows logistic regression analyses of the association between patients’ demographics, clinical characteristics and prevalence of bacterial infection. None of the factors was significantly associated with prevalence of bacterial ocular infections (p > 0.05).

**Table 5:** Factors associated with external ocular and periocular infections among a Ghanaian ophthalmic population.

Variable	Bivariate regression		p-value
	OR	95% CI	
***Demographic characteristics***			
Age (years)	1.04	0.97–1.11	0.237
**Age group (years)**			
0–2	Ref		
3–17			0.998
18–39			0.999
≥ 40	1.00		1.000
**Gender**			
Man	Ref		
Woman			0.998
Not applicable*			0.998
**Sex designated at birth**			
Male	Ref		
Female	0.32	0.03–2.97	0.316
**Ethnicity**			
Akan	Ref		
Northerner			0.999
**Religion**			
Catholic	Ref		
Protestant			0.999
Muslim	1.00		1.000
Atheist	1.00		1.000
***Socioeconomic characteristics***			
**Residence**			
Rural	Ref		
Urban	0.99	0.16–6.21	0.993
**Highest level of education**			
None	Ref		
Preschool	1.00		1.000
Primary			0.999
Secondary			0.999
Tertiary	1.00		1.000
Not applicable*	1.00		1.000
**Occupation**			
Not applicable*	Ref		
Student			0.999
Trading	1.00		1.000
Welding	1.00		1.000
Hairdressing	1.00		1.000
Unemployed	1.00		1.000
Construction worker	1.00		1.000
Beautician	1.00		1.000
Wage or salary worker	1.00		1.000
Driver/Transport business	1.00		1.000
Businessman/woman	1.00		1.000
Farming	1.00		1.000
Haidressing	1.00		1.000
**Marital status**			
Not applicable*	Ref		
Single	2.24	0.24–21.29	0.481
Married			0.998
Cohabiting			0.999
Divorced			0.999
Seperated			1.000
Widow			0.999
***Health status variables***			
**Smoking habits**			
Not applicable*	Ref		
Never smoker	6.27	0.67–58.30	0.107
Past smoker			0.999
Current smoker			1.000
**Alcohol intake habits**			
Not applicable*	Ref		
I never drink	5.67	0.61–52.83	0.128
I drink only on special occasions			0.999
I drink once or twice a week			0.999
**Average alcohol consumption per week**			
None	Ref		
1 unit			0.999
2–5 unit			0.999
***Medical history***			
**Diabetes**			
No	Ref		
Yes			0.999
**Hypertension**			
No	Ref		
Yes			0.999
**Tuberculosis**			
No	Ref		
Yes			1.000
**Sexually transmitted diseases**			
No	Ref		
Yes			1.000
**Peptic ulcer**			
No	Ref		
Yes			0.999
**Are you currently taking any medication**			
No	Ref		
Yes			0.998
**Clinical characteristics**			
**Eyes affected**			
Right eye; oculus dexter	Ref		
Left eye; oculus sinister	0.47	0.04–5.70	0.555
Both eyes; oculus uterque	1.75	0.15–20.42	0.655
***Site of the eye affected***			
**Eyelids/lashes**			
No	Ref		
Yes			0.998
**Conjunctiva**			
No	Ref		
Yes			0.999
**Cornea**			
No	Ref		
Yes	0.36	0.06–2.31	0.282
***Risk factors***			
**Previous eye infections**			
No	Ref		
Yes	0.19	0.02–1.77	0.145
**Previous use of antimicrobials**			
No	Ref		
Yes			0.998
**Previous use of contact lens**			
No	Ref		
Yes			1.000
**Previous use of spectacles**			
No	Ref		
Yes	0.46	0.05–4.53	0.505
**Previous ocular trauma**			
No	Ref		
Yes			0.998
**Previous ocular surgery**			
No	Ref		
Yes			0.999
**Duration of illness**			
< 1 week	Ref		
2–4 weeks			0.998
> 4 weeks	1.22	0.13–11.62	0.863
**Previous application of mascara**			
No	Ref		
Yes			0.999
**Previous application of breastmilk**			
No	Ref		
Yes			0.999
***Patient presenting symptoms***			
**Eye pain**			
No	Ref		
Yes	0.29	0.03–2.73	0.294
**Itching**			
No	Ref		
Yes	2.88	0.31–26.70	0.352
**Falling lashes**			
No	Ref		
Yes			1.000
**Lacrimation/watering**			
No	Ref		
Yes	1.10	0.18–6.89	0.920
**Hyperemia/redness**			
No	Ref		
Yes	0.63	0.07–5.84	0.680
**Swelling**			
No	Ref		
Yes			0.998
**Photophobia**			
No	Ref		
Yes	0.32	0.05–2.03	0.315
**Discharge**			
No	Ref		
Yes	2.27	0.36–14.21	0.381
**Burning sensation**			
No	Ref		
Yes			0.999
**Foreign body sensation**			
No	Ref		
Yes			
**Others**			0.999
No	Ref		
Yes	0.56	0.06–5.42	0.615

Bivariate regression analysis at a significance of p < 0.05.

*OR* odds ratio, *CI* confidence interval, *Ref* reference.

The novel study for the first time aims to investigate the bacteria etiology of external ocular and periocular infections and antimicrobial treatment patterns among a Ghanaian ophthalmic population. About 95% of the culture were positive for bacteria pathogens, and with the predominant class of bacteria being Gram negatives. *Pseudomonas aeruginosa* and *Staphylococcus aureus* were the commonly isolated bacteria species and with the former frequently isolated in cases of conjunctivitis and keratitis. The commonly used antimicrobial therapy in the clinical management of eye infections in these facilities were polymyxin B, neomycin and ciprofloxacin.

Bacteria ocular and periocular infections pose health challenges owing to associated morbidity and blindness. Globally, the burden of bacteria eye infections is higher especially in lower-and-middle income countries including Ghana^2–4^. Though microscopic, the wide biodiversity of bacteria pathogens makes it burdensome for ophthalmic clinicians and physicians when selecting appropriate antibiotic therapy in routine clinical management of ocular and periocular infections. Previously, authors from several geographical jurisdictions have investigated the burden and etiology of bacteria eye infections, however, outcomes from these studies varied considerably^13–15,18,20,25,26^. The prevalence estimates of medical conditions such as bacteria ocular and periocular infections are critical in informing eye care service delivery and in the development of policies to strengthen eye care practices yet there is presently limited ophthalmic data to propagate such transitions within the Ghanaian context. Importantly, gaining insight on bacteria etiology implicated in cases of external ocular and periocular infections within the Ghanaian population is essential to guide clinicians in the appropriate choice of antimicrobial therapy. Nonetheless there is paucity of data in this regard.

Overall, the prevalence of bacteria ocular and periocular infections found in this study was 95.1%. Our results are comparable with studies in Ethiopia^50^, Saudi Arabia^51^, Italy^25^ and United States of America^26^. In a cross-sectional study in Ethiopia, Tesfaye et al. reported a prevalence of 74%^50^. Similarly, a study by Shahaby and colleagues utilizing participants from a university clinic in Saudi-Arabia found more than two thirds of ocular specimens harboring bacteria pathogens^51^. Likewise, an observational case series conducted in Italy by Papa and coworkers, revealed that the proportion of bacterial infections was estimated at 72.5%^25^. Furthermore, in a prospective observational study among patients undergoing cataract surgery in the U.S.A., Ta et al. showed that almost eight of every ten ocular specimen obtained from patients eyes had a bacteria etiology^26^. On the one hand, estimates from the present study is significantly higher and varies substantially compared to studies in China^13,18^, Iran^20^, South Korea^14^ and Nepal^15^ with prevalence estimates far lower than 50%^13–15,18,20^. Although geographical settings, study population, seasonality and laboratory procedures could account for such variations as reported earlier, a plausible reason for our observation may be attributable to the fact that our study unlike previous investigations enrolled patients from multiple eye care facilities hence the burden of infections maybe summative. Another reason is that majority of our study participants were rural dwellers with sanitation in such areas usually problematic compared to inhabitants in urban vicinities.

We observed a slightly higher proportion of Gram-negative bacteria compared to Gram positives as etiological agent in our study. This findings contrast with studies from China^13^, Ethiopia^11^, Saudi-Arabia^16^, Uganda^4^ and United States of America^26^ where Gram negatives were found to be significantly lower compared to Gram positive bacteria and with proportional estimates of ranging from six to ten percent^4,11,13,16,26^. Conversely, the proportionate distribution of Gram negatives to positive bacteria found in this study are parallel with results from several existing literatures^2,5,8,10^. For example, among the various ocular microbiology investigations conducted across Ethiopia, by Ayebubizu et al.^5^, Belyhun et al.^2^, Assefa et al.^10^ as well as Getahun and colleagues^8^ the proportionate distribution of Gram-negatives were similar to Gram positives. Of note, whereas the magnitude of Gram-negative bacteria etiology found in Australia^21^, Iran^20^ and Italy^24^ were not equivalent to our findings as well as studies in parts of Ethiopia, the proportion estimates reported were relatively higher. There exist regional variations in the patterns of distribution of Gram-negative bacteria, however, the higher prevalence in our study are ascribed to hygiene as the primary mode of transmission of these enteric bacteria are through oral-fecal contamination. Specifically, we observed during data collection that most patients repeatedly clean ocular discharges with either bare hands or face handkerchief, hence predisposes eyes to contamination by fecal contaminants. Additionally, majority of our study subjects were either in preschool and/or primary hence prone to eye contamination through outdoor gaming activities in school. A considerably higher proportion of the study participants were below two years, and these age categories frequently experience oral-ocular contamination through inserting hands in mouth and touching of eyes thereafter which may have accounted for the increasingly abundance of Gram negative bacteria than positives in our study.

The predominant bacteria species found in our study were *Pseudomonas aeruginosa* and *Staphylococcus aureus*. Although, *S. aureus* was second only to *P. aeruginosa* as the frequently isolated bacteria pathogen, however, it remains the most abundant Gram positive bacteria isolate from all obtainable ocular specimen in our study. This finding are consistent with studies in India^19^, Italy^24^, Nigeria^52^, and Ethiopia^4,8,12^. The occurrence of ocular infections with *S. aureus* etiology may be due to frequent touching of eyes with filthy hands among study subjects. The incidence of *Pseudomonas aeruginosa* in eye infections are mostly linked with the wearing of contact lens nonetheless we observed an inverse trend in our study. The blinding risk factor associated with *Pseudomonas aeruginosa* ocular infections underscores the promotion of contact lenses as an alternative to spectacle glasses in vision correction and/or cosmesis. Importantly *Pseudomonas aeruginosa* are opportunistic pathogens with devastating consequences on the ocular tissues. Specifically, they induce cornea infiltration and ulcerative keratitis when improperly managed by clinicians. Further, the conjunctiva and cornea are in close proximity landmarked by the limbus, hence pathogens of the conjunctiva can easily spread to the cornea during physiological blinking or mechanical rubbing of the eyes. Given the predominance of *Pseudomonas aeruginosa* in cases of conjunctivitis and keratitis warrants the need for clinicians to probe for other proxy predisposing factors other than relying solely on contact lenses etiology in most instances.

The commonly administered antimicrobial therapy found in this study were Polymyxin B, neomycin and ciprofloxacin. Polymyxin B is a nonribosomal peptidic antimicrobial agents used mostly in the treatment of Gram-negative infections. In particular, they exert their bactericidal effect by binding to phosphate residues within the lipopolysaccharides cell wall to induce displacement of divalent magnesium and calcium cations known to maintain membrane stabilizing properties of Gram-negative bacteria. Consequently, the intrinsic mechanism of action primarily causes an increase in cell membrane permeability resulting in a direct loss of cytoplasmic cell contents. Furthermore, they act synergistically with beta-lactam antibiotics by exposing the peptidoglycan machinery of these Gram negatives for which the latter act on^53^. On the contrary as an aminoglycoside neomycin actively inhibits protein synthesis of bacteria by insurmountably binding to the 16S ribosomal R.N.A. as well as 50S ribosomal subunits of susceptible class of Gram bacteria^54,55^. Similarly, ciprofloxacin a fluoroquinolone prevents bacteria D.N.A. replication by terminating the action of the reaction enzymes D.N.A. topoimerase IV and D.N.A. gyrase. The ensued effect is suicidal against Gram negatives as well as mixed bacteria culture^56^. Altogether, the frequent use of the aforementioned antibiotic agents in clinical management of ocular and periocular eye infections in our study are concordant with the laboratory results which identified Gram negatives as the predominant bacteria isolates.

The previous use of antimicrobials among patients is usually considered a risk factor in ocular infection due to the increased potential for contamination from improper handling or storage^8,57^. Although Getahun et al.^8^ in northwestern Ethiopia reported a significant association between the previous use of antimicrobials and the presence of positive bacterial culture, our results showed otherwise. Thus, patient characteristics, such as the prior usage of antimicrobials, were not significant determinants of positive bacterial culture. This was consistent with a similar investigation by Belyhun and coworkers^57^. Considering the varying resistance mechanism of microorganisms to single antimicrobial therapy and its negative repercussion in resistant eye infections, the administration of two/more specific antibiotic treatment underscores the patronage of a single broad-spectrum antibiotic, an essential determinant in antimicrobial resistance.

Of note, the study has several strengths worth highlighting. The study presents a preliminary and most recent data on bacterial etiology of external ocular and periocular infections among ophthalmic patients in Ghana. Although, we recommend future ocular antibiotic sensitivity studies in this setting, however, in light of the present evidence on the bacterial isolates implicated in eye infection are essential in assisting ophthalmic clinicians in their choice of antibiotic therapy. Moreover, unlike previous studies^2,7,58^ the present investigation utilized sample from multiple sites which underscores the selection bias usually associated with the convenience sampling approach which the study employed. On the contrary, owing to resource limitation the study could not performed direct fluorescent antibody test and/or Giemsa staining to investigate infections of *Chlamydia trachomatis* etiology. Our prevalence may have been underestimated as a considerable number of our patients where preschoolers whose uncooperative nature denied researchers from taking ocular swabs for bacteriological analyses. Although, given the nature of the studies we could not ascertain the treatment outcomes of the patients the information on the therapy was pivotal in our laboratory.

## Conclusion

The prevalence of positive bacteria culture from external ocular and periocular infections was approximately 95%. Gram-negative organisms were commonly implicated and with *Pseudomonas aeruginosa* and *Staphylococcus aureus* as the predominant causative bacteria. Clinical presentations of conjunctivitis and keratitis infections were mostly caused by *Pseudomonas aeruginosa* and with polymyxin B, neomycin and ciprofloxacin as the frequently administered antimicrobial therapy. Given the high burden of ocular bacterial infections, measures (infections control program and antimicrobial agent management program) should be institutionalized to prevent emergence of resistant strains. We recommend future studies to focus on investigating into the potential antibiotic resistances infections within the Ghanaian ophthalmic population.

## Methods

### Study design, setting and population

A multi-center study was conducted among patients suspected of external ocular and periocular infections in three health facilities in Ghana, namely Anglican Eye Hospital, Jachie; St. Michaels Hospital, Pramso; and Kumasi South Hospital, Agogo from July 18, 2021 to September 18, 2021. Cornea scrapings and conjunctival specimens were obtained from infected eyes for bacterial isolation together with collation of patients sociodemographic and clinical characteristics with a pretested structured questionnaire.

### Study setting

The Anglican Eye Hospital (A.E.H.), St. Michaels Hospital (S.M.H.) and Kumasi South Hospital (KSH) were selected for the study primarily because of their higher out-patient-department (O.P.D.) turnout, as well as their wide catchment area and rural/urban interactions. The A.E.H. and S.M.H. are located in Bosomtwe; a rural district in Ghana whilst KSH is situated in the Asokwa municipal area, an urban settlement in the Ashanti Region of Ghana. All the facilities have either a permanent/visiting ophthalmologist, optometrists, ophthalmic nurses and opticians. All the facilities provide comprehensive eye services which range from case history, visual acuity assessment, refraction, dispensing of refractive glasses, management of anterior and posterior segment pathologies, prescribing of medications and performing scheduled surgeries. The S.M.H. and KSH, serve as immediate referral hospitals for the surrounding private and polyclinics. However, all clinical emergencies are referred to the Komfo Anokye Teaching Hospital, the only tertiary health facility in the region. The study facilities lack microbiology laboratory hence clinicians employ empirical approaches in their routine diagnosis and management of external ocular and periocular infections.

### Study population and sampling

The study population involved patients who sought ophthalmic treatments/ services for external ocular and periocular infections at the eye clinics of the Anglican Eye Hospital, Jachie; St Michaels Hospital, Pramso; and the Kumasi South Hospital from July 18 to September 18, 2021. Purposive sampling approach was used to recruit all patients presenting with signs and symptoms of external ocular and periocular infections following a consent (and accent for minors). Patients reporting solely for optical correction, or participants on systemic/topical antibiotics or have performed ocular surgery in the last one week were excluded. A purposive sampling approach was used to recruit all one hundred and fourteen (114) eligible subjects.

### Study variables

The independent variables for this study were participants’ sociodemographic factors; age, sex, ethnicity, religion, facility, socioeconomic; residence, highest level of education, occupation, marital status, health status; smoking habits, alcohol intake, systemic medical conditions and clinical characteristics (eyes affected, site of the eyes affected, risk factors, patient presenting symptoms) whereas the outcome/dependent variable was prevalence of bacteria ocular and periocular infections. Participants were assessed and clinical presentations classified based on operational terms reported previously^7^, and antimicrobial treatments documented accordingly.

### Operational definitions

Blepharitis in the study was characterized by gritty itchy sore eyes, with crusting and/or collarattes around the base of the eyelashes coupled with clogging of the Meibomian gland, loss of eyelashes and with demodex conjunctivitis. Conjunctivitis was defined as conjunctival lesion delineated by hyperemia, chemosis, whitish tint purulent discharge and hemorrhage. Blepharoconjuntivitis presented as redness of the eye, with dry scaly eyelids and ensuing symptoms of itchiness and burning sensation. In keratoconjuntivitis, cornea and conjunctiva were implicated with complaints of dryness, itching and mucous discharge. Keratitis was defined as lesion of the cornea with characteristic cornea edema, cellular infiltration, pain, redness, photophobia and ciliary injection. Hordeolum was defined swelling and tenderness of the eyelid with acute pain, photophobia and mild epiphora. Ophthalmia neonatorum is a neonatal conjunctivitis presented within the first 28 days of life with signs of eyelid edema, erythema and purulent discharge. Preseptal and orbital cellulitis showed similar features of painful swelling and/or tenderness of the eyelid with the later distinguished from the former by decreased vision and pain on eye motility.

### Data collection

#### Sociodemographic and clinical data

The patients sociodemographic, socioeconomic, and health status variables were gathered by the principal investigator and a trained research assistant using a pre-tested structured questionnaire. A comprehensive vision assessment including visual acuity, slit lamp biomicroscopy, and ophthalmoscopy was performed by a registered optometrist on all study participants. Subsequently, patient medical history, primary and secondary diagnosis, and antimicrobial therapy prescribed were extracted using a data collection form.

#### Specimen collection and transport

Overall, 103 ocular specimens were obtained from the eyes of patients with external ocular and periocular infections following aseptic procedures. With the patient eyes in an upward position of gaze, conjunctival specimens were obtained by gently rolling a moistened saline cotton bud over the lower tarsal plate of the eyelids and fornix of the conjunctiva in repeated strokes, thus from nasal to temporal and vice versa. Samples from corneal ulcer and keratitis infections were obtained utilizing a modified version of an original protocol described previously^8^. Briefly, using slit-lamp biomicroscopy and under topical local anesthesia (1–2 drops of 0.5% fresh proparacaine), the edges of the ulcer were firmly scraped^8,9,19^. On the contrary, none of the patients in our study presented with dacryocystitis and/or blepharitis; hence a puncture and/or aspiration of the lacrimal sac as well as swabbing on the eyelids were not undertaken. The swabs were subsequently kept in a sterile, freshly prepared nutrient broth and transported within 1–3 h in a standard triple packaging system (of an absorbent cotton wrapping primary container enclosed in a sealed bag and kept in an insulated icebox with icepacks) from the study sites to the Microbiology Laboratory of the Faculty of Pharmacy and Pharmaceutical Sciences, Kwame Nkrumah University of Science and Technology, for further microbial investigation.

### Laboratory methods

#### Culture and identification of bacteria pathogens

**Figure 1:**
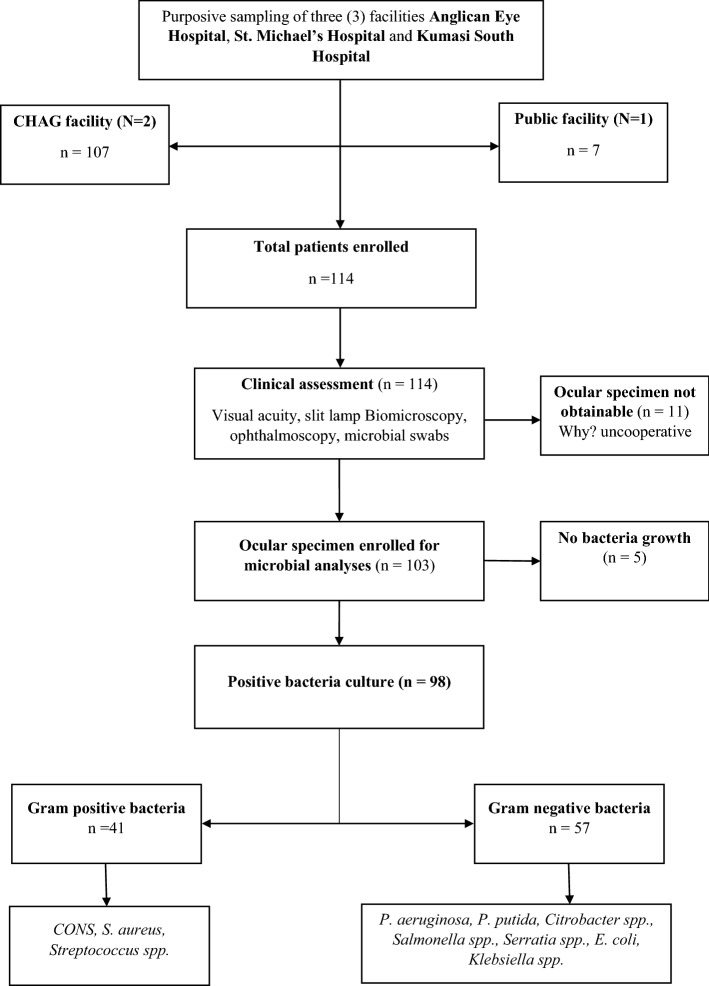
Flow diagram illustrating study methodology.

All specimen obtained were initially inoculated on a Nutrient agar and incubated at 37 °C for 24 h and plates examined for growth afterwards. Plates with microbial growth were transferred onto various differential and selective media for preliminary isolation and identification. Specifically, bacteria were cultured on a Mannitol Agar, MacConkey Agar, Bismuth sulphite (all from Oxoid Ltd. Basingstoke, Hants United Kingdom brand), Cetrimide agar (HiMedia Laboratories Pvt. Ltd Mumbai, India), 5% sheep Blood and Chocolate agars and incubated for 24 h. Of note, with the exception of 5% blood and chocolate agars which were kept under anaerobic conditions, an aerobic atmosphere was maintained for all other differential agar media used. Conversely, plates with no growth were re-incubated for additional 48 h and consequently counted negative if no growth pattern appeared. In addition, microbial growth on differential media was again sub-cultured on a nutrient agar to obtain pure colonies and subsequently subjected to further phenotypic identification, specifically colony morphology, Gram stain, and biochemical analyses. Gram-positive bacteria isolates were characterized using coagulase, catalase, and bacitracin tests, whereas Gram-negative bacteria were differentiated using citrate utilization, lysine decarboxylase agar, indole, and urease tests, as well as triple sugar iron agar^31,59,60^. Details of the methodology are summarized in Fig. 1.

#### Quality assurance and control

The questionnaires employed to gather the sociodemographic and clinical data were pretested at the Anglican Eye Hospital and revised accordingly following their feedback. Questionnaires were written in English and administered by the principal investigator and a trained research assistant. On the one hand, questionnaire was explained in local dialect for study subjects who could not comprehend instructions in English language. The data from study was doubled checked for accuracy and completeness. At the facility, all test tubes with ocular samples were well-labeled to avoid any mismatched. The laboratory reagents and culture media for the experiments were checked for expiry dates, and sterility control performed to ascertain the integrity of the media such as free from contamination. The media performance and/or functionality assessment was conducted using the American Type Culture Collection (A.T.C.C.) Standard Reference Strains. Specifically, *Escherichia. coli* ATCC 25,922; *Pseudomonas aeruginosa* ATCC 4853; *Staphylococcus aureus ATCC* 25,923.

### Data protection and management

The hardcopy version of the filled questionnaires was kept under lock and key and accessible only to the principal investigator and research advisor. Similarly, the softcopy of the non-aggregated dataset was protected using an alphanumeric stringed password. The research report presented to the respective facilities and for publication purposes were aggregated hence individual study participants could not be traced.

### Ethical consideration

To undertake this study a hierarchical ethical consideration protocols were followed. A written permission was sought from the authorities of the Anglican Eye Clinic, St. Michaels Hospital, and Kumasi South Hospital. The study protocol was then approved by the Committee on Human Research, Publication and Ethics (C.H.R.P.E.), of the Kwame Nkrumah University of Science and Technology and the Komfo Anokye Teaching Hospital (Reference number: CHRPE/AP/282/21). Written informed consent was obtained from adult participants and for minors a written informed consent was taken from caregivers after study protocol was fully explained to the best of their comprehension. The study adhered to the tenets of the declaration of Helsinki^61^, and all laboratory procedures performed in accordance to the Clinical Laboratory Standard Institute guidelines, C.L.S.I.^62^.

### Statistical analysis

Data were entered and managed in Microsoft Excel and further exported into Statistical Package and Service Solution version (I.B.M. Corporation IBM® SPSS® Statistics for Windows, version 25.0 Armonk, NY) compatible with windows. Normality assessment was performed using the Kolmogorov Smirnov statistic. The demographics, socioeconomics, health status and clinical characteristics of the sample were presented, and the difference between males and females was tested with chi-square analysis. Clinical diagnosis, cultural status and antimicrobial treatments were presented in cross tabulations using frequencies and percentages. Association between sample characteristics and prevalence of bacterial infections were investigated using bivariate logistic regression at a significance set at p < 0.05 (Supplementary Information S1).

## Supplementary Information

Supplementary Information.

## Data Availability

All relevant data and materials supporting the conclusion of this article is/are available within the manuscript and its supporting information files.

## Abbreviations

A.E.H.: Anglican Eye Hospital
A.M.R.: Antimicrobial resistance
ARMOR: Antibiotic resistance monitoring in ocular microorganisms
C.H.A.G.: Christian Health Association of Ghana
C.H.R.P.E.: Committee on human research publication and ethics
C.I.P.: Ciprofloxacin
C.L.S.I.: Clinical laboratory standard institute guidelines
CONS: Coagulase negative Staphylococci species
C.O.R.: Crude odds ratio
D.N.A.: Deoxyribonucleic acid
FX: Flucoxacillin
KSH: Kumasi South Hospital
G.T.M.: Gentamycin
M.D.R.: Multi drug resistance
R.F.R.: Referral
PoB: Polymyxin B
NM: Neomycin
TBM: Tobramycin
TX: Tetracycline
O.X.T.: Oxytetracycline
OFC: Ofloxacin
R.N.A.: Ribonucleic acid
S.M.H.: St Michaels Hospital
